# Genetic landscape and molecular targets in pediatric pineal tumors

**DOI:** 10.1093/noajnl/vdag086

**Published:** 2026-04-03

**Authors:** Elena Pasquinelli, Milena Guidi, Iacopo Sardi

**Affiliations:** Neuro-Oncology Unit, Cancer Genetics Program, Meyer Children’s Hospital IRCCS, Florence, Italy; Neuro-Oncology Unit, Cancer Genetics Program, Meyer Children’s Hospital IRCCS, Florence, Italy; Neuro-Oncology Unit, Cancer Genetics Program, Meyer Children’s Hospital IRCCS, Florence, Italy

## Abstract

Pineal region tumors are rare and biologically heterogeneous central nervous system neoplasms that occur predominantly in the pediatric population and are associated with significant morbidity and mortality. Among these, pineal parenchymal tumors encompass a spectrum of entities ranging from indolent pineocytomas to highly aggressive pineoblastomas. Recent advances in genomic, epigenomic, and transcriptomic profiling have fundamentally reshaped the understanding of these tumors, moving beyond purely histological classification toward molecularly defined subgroups with distinct biological behavior and clinical outcomes.

This review provides a comprehensive overview of the current molecular landscape of pineal region tumors, with a particular focus on genetic predisposition, somatic driver alterations, DNA methylation profiles, and transcriptional programs across pineocytoma, pineal parenchymal tumor of intermediate differentiation (PPTID), pineoblastoma, papillary tumor of the pineal region (PTPR), and desmoplastic myxoid tumor, *SMARCB1*-mutant. Key oncogenic mechanisms involving microRNA biogenesis disruption, cell-cycle deregulation, MYC/FOXR2-driven transcriptional amplification, PI3K/AKT/mTOR pathway activation, and chromatin remodeling defects are discussed, highlighting their prognostic and therapeutic relevance.

In particular, the molecular subdivision of pineoblastoma into distinct subgroups has revealed subgroup-specific vulnerabilities that may be exploitable through targeted therapies. Emerging translational approaches, including molecularly guided treatment strategies and rapid intraoperative sequencing technologies, are also addressed. Despite these advances, the rarity of pineal region tumors continues to limit large-scale clinical trials. Multicenter collaboration and systematic integration of molecular profiling into clinical practice will be essential to improve outcomes for affected children.

Key Points

- Molecular profiling defines subgroups in pediatric pineal tumors.
- Genetic and epigenetic drivers reveal targets for precision therapy.
- Lineage transcriptional programs influence tumor biology and prognosis.

Importance of the Study

Pineal region tumors in children are rare, heterogeneous, and associated with significant morbidity and mortality, yet their biological drivers and therapeutic vulnerabilities remain incompletely understood. This review provides a comprehensive synthesis of current knowledge on germline predisposition, somatic alterations, epigenetic subgroups, and transcriptional programs underlying pediatric pineal parenchymal tumors. By integrating genomic, methylation, and transcriptomic data, we highlight how molecular profiling is reshaping tumor classification, risk stratification, and treatment selection beyond conventional histology. We also summarize emerging targeted and pathway-based therapeutic strategies linked to specific molecular subtypes. This work offers a unified framework for clinicians and researchers to interpret molecular findings in clinical practice and trial design. Ultimately, it aims to support the development of precision medicine approaches and to improve outcomes for children affected by these rare and challenging brain tumors.

Pineal region tumors represent a rare but clinically significant subset of pediatric central nervous system (CNS) neoplasms, accounting for approximately 3%-11% of all pediatric brain tumors, compared to less than 1% in adults.^1^ Within this group, pineal parenchymal tumors, including pineocytoma, pineal parenchymal tumor of intermediate differentiation (PPTID), pineoblastoma (PB), papillary tumor of the pineal region (PTPR), and desmoplastic myxoid tumor SMARCB1-mutant, constitute roughly 20%-30% of lesions arising in this anatomical location, while germ cell tumors remain the most frequent histological type.^2^ Pediatric PBs, classified as WHO grade 4 neoplasms, display aggressive biological behavior with a high tendency to leptomeningeal dissemination and dismal prognosis despite multimodal approach therapy. In contrast, low-grade pineal parenchymal tumors such as pineocytomas are rarer in children but associated with more favorable outcome.^3^ Across reported series, the distribution of pineal parenchymal tumors in children includes approximately 12% PBs, 12% pineocytomas, and 10%-20% PPTID.^2^ Clinical presentation is typically dominated by signs of increased intracranial pressure, including headache, vomiting, papilledema, and hydrocephalus, as well as visual dysfunction due to dorsal midbrain compression.^4^ Reported 5-year overall survival rates range from 39% to 79%, with poorer outcomes associated with higher-grade histologies and metastatic disease at diagnosis.^3^ Despite their rarity, these tumors carry significant morbidity and mortality, highlighting the urgent need for improved molecular characterization and targeted therapeutic strategies.

Diagnosis relies primarily on magnetic resonance imaging (MRI) for lesion detection and characterization, combined with histopathological examination following surgical biopsy or resection, which remains essential for tumor classification and grading.^5^ Molecular tools, including DNA methylation profiling, are increasingly used to refine tumor classification and to distinguish aggressive forms such as PB from other histotypes.^6^ Current therapeutic standards are based on maximal safe surgical resection, which provides tissue for diagnosis and alleviates hydrocephalus when present, followed by tailored adjuvant therapy depending on histology and staging. For aggressive tumors like PB, treatment typically combines standard and high- dose chemotherapy associated with craniospinal radiotherapy, whereas low-grade tumors may be managed with surgery alone or less intensive adjuvant therapy.^7^^,^^8^

Recent years have witnessed a growing emphasis on the role of genetics and molecular profiling in CNS tumors, revolutionizing tumor classification, prognostic stratification, and treatment decision-making.^9^^,^^10^ Advances in next-generation sequencing (NGS) technologies have uncovered the complex genomic and epigenomic landscape of brain tumors, revealing recurrent somatic driver mutations, germline predisposition syndromes, and novel therapeutic targets.^11^ Despite this progress, the integration of molecular data into real-time clinical workflows, especially in pediatric CNS tumors, remains limited compared to adult oncology.^12^ Innovative approaches, such as intraoperative sequencing platforms like the Oxford Nanopore-based “ROBIN” system, are emerging to enable rapid genomic characterization during neurosurgical procedures, potentially allowing immediate surgical and therapeutic adjustments.^13^ However, pediatric pineal region tumors, due to their rarity and heterogeneity, are underrepresented in genomic studies, leading to a significant knowledge gap regarding their genetic drivers and clinical implications. A comprehensive review synthesizing current knowledge on genetic alterations, molecular biomarkers, methylation profiles, small RNAs, and gene-targeted therapies of these rare tumors is crucial. Such a resource could not only guide future research but also inform precision medicine approaches in pediatric neuro-oncology, where treatment options are still largely based on histology rather than molecular features.^6^^,^^7^

## Pineocytoma

Pineocytoma is a well-differentiated pineal parenchymal neoplasm that typically exhibits slow, locally confined growth and remains restricted to the pineal region. It accounts for approximately 25% of all pineal parenchymal tumors. Pineocytomas can occur across a wide age range but most frequently affect adults, with a median age at diagnosis of around 44 years and show a consistent female predominance. Prognosis is generally favorable, with reported 5-year overall survival rates ranging from 86% to 91%.

From a molecular perspective, pineocytomas appear to be genetically stable tumors. Large sequencing cohorts have not identified recurrent single-nucleotide variants, including canonical *DICER1* or *KBTBD4* point mutations, supporting a predominantly epigenetic mode of tumorigenesis^14^^,^^15^ (Table 1). Consistently, cytogenetic abnormalities are rare, with most reported cases displaying near-diploid karyotypes and no recurrent copy-number alterations.^16^ This genomic stability is in keeping with the relatively indolent clinical behavior of pineocytomas when compared with high-grade pineal parenchymal tumors such as PB. On DNA methylation profiling, cases diagnosed as pineocytoma cluster within a distinct epigenetic subgroup located in close proximity to normal pineal gland tissue, further supporting their well-differentiated biological identity^17^ (Figure 2).

**Table 1.** Germline and somatic genetic alterations in pineal region tumors

Pineal tumor type	Constitutive alterations	Tumor alterations
Pineocytoma	**Pallister-Killian syndrome** (mosaic tetrasomy 12p; *anecdotal/low evidence*)^18^ ^e^	No recurrent SNVs reported; generally **genomically stable** (no consistent CNAs).^14–16^ ^a^ ^b^
PPTID (Pineal parenchymal tumor of intermediate differentiation)	***DICER1*** (*rare/low evidence*)^22^ ^f^	Recurrent ***KBTBD4*** in-frame insertions; ***ATRX*** loss/inactivation (subset; ALT); occasional alterations reported: ***TSC1***, ***PTEN***, ***TP53*** (often via CN loss rather than SNV).^23^ ^,^ ^29^
Pineoblastoma (PB)	***RB1***, ***DICER1***, ***DROSHA***, ***DGCR8***; less frequent: ***APC*** (Turcot), ***NSD1*** (Sotos; isolated report)^43–51^	Subgroup drivers: ***DICER1/DROSHA/DGCR8*** (biallelic LOF; miRNA subgroups), ***RB1*** (biallelic inactivation; PB-RB1), ***MYC*** amplification + ***FOXR2*** activation (PB-MYC/FOXR2); recurrent CN gains incl. ***PDE4DIP*** (duplications/gains, enriched in aggressive subtypes); frequent CN patterns (eg chr14 loss).^52–55^ ^c^ ^d^
PTPR (Papillary tumor of the pineal region)	***PTEN*** (PTEN hamartoma tumor syndrome; early-onset subset)^73^	***PTEN*** loss-of-function (truncating mutations/deletions; often exon 7/C2 domain); frequent **chromosome 10 loss** (PTEN locus); recurrent CNAs: losses chr3/14/22; gains chr8p/9/12^77^ ^,^ ^80^ ^,^ ^81^
DMT, *SMARCB1*-mutant (Desmoplastic myxoid tumor of pineal region)	None established	Biallelic ***SMARCB1*** inactivation (homo/heterozygous deletions + second hit frameshift/duplication); recurrent **22q loss** (SMARCB1 locus)^86^

a SNV, single-nucleotide variant

b CNA, copy-number alteration

c LOF, loss of function

d CN, copy number

e Constitutive alterations and relevant Tumor alterations are highlighted in bold

f Gene symbols are presented in italic, in accordance with standard nomenclature

Rare associations with genetic syndromes have been reported. To date, only 2 patients with Pallister-Killian syndrome (PKS) and pineal lesions have been described in the literature, both based exclusively on neuroradiological findings without histopathological confirmation. More recently, the first case of a histologically confirmed pineocytoma in a patient with PKS has been reported, representing the first documented association between this chromosomal mosaicism and a pineal parenchymal tumor. PKS is a rare sporadic chromosomal disorder caused by mosaic tetrasomy of the short arm of chromosome 12 [i(12p)], resulting from the presence of a supernumerary isochromosome in a subset of somatic cells (Table 1). Although this observation remains anecdotal, it raises the possibility that mosaic genomic imbalances involving chromosome 12p may contribute to tumor susceptibility in selected tissues, warranting further investigation.^18^ Given the lack of recurrent actionable genetic drivers, targeted therapies currently have no established role in pineocytoma management. Future studies integrating multi-omic profiling may clarify whether rare molecular subsets could benefit from precision approaches.

## Pineal Parenchymal Tumor of Intermediate Differentiation

Pineal parenchymal tumor of intermediate differentiation is a pineal parenchymal neoplasm with an intermediate degree of malignancy between pineocytoma and PB. PPTIDs arise in the pineal region and frequently demonstrate local invasive growth.^19^ Although generally less aggressive than PBs, PPTIDs retain the potential for local recurrence and, in some cases, craniospinal dissemination.^7^^,^^9^ PPTIDs account for approximately 45% of pineal parenchymal tumors.^20^ In a pooled analysis of 29 studies, including 127 patients with PPTID, the median overall survival was 14 years, with a reported 5-year overall survival rate of 84%.^21^

To date, no strong syndromic associations or inherited genetic susceptibilities have been consistently reported in PPTID. DICER1 syndrome is a rare inherited tumor predisposition syndrome associated with an increased risk of several malignant and benign neoplasms. A patient with PPTID harboring a germline pathogenic variant in the *DICER1* gene has been described. While PB is a well-established DICER1-related tumor, the association between PPTID and *DICER1* mutations remains rare, with only one recent large molecular study reporting this finding^22^ (Table 1).

From a molecular standpoint, PPTIDs are most frequently driven by recurrent small in-frame insertions in *KBTBD4*, typically involving exon 2 within the Kelch domain hotspot, such as c.152_153insGAA (p.Ser51dup) or c.155_156insAAG (p.Asn52dup), which disrupt substrate recognition.^17^ In a subset of PPTIDs, biallelic inactivation of *ATRX* has been reported, including nonsense variants such as c.6835C>T (p.Arg2279*), leading to loss of ATRX protein expression and activation of the alternative lengthening of telomeres (ALT) phenotype^23^ (Table 1).

These findings highlight mechanistic diversity within intermediate-grade pineal parenchymal tumors, implicating both the ubiquitin-proteasome system and telomere maintenance pathways.

*KBTBD4*, a BTB-Kelch adaptor for CUL3-RING ubiquitin ligases, mediates aberrant ubiquitin-dependent proteolysis, altering the turnover of key regulatory proteins.^17^ The recurrent in-frame insertions affecting the Kelch domain in PPTID disrupt substrate recognition, leading to altered proteostasis and accumulation of aberrant proteins. On this basis, pharmacological inhibition of the proteasome may be proposed as a potential strategy to exacerbate proteotoxic stress in KBTBD4-mutant tumors.^24^ Proteasome inhibitors have shown activity in preclinical models of pediatric CNS tumors and have been tested in early-phase CNS trials; their use has not been reported nor validated specifically in KBTBD4-mutant pineal tumors.^25^ More recently, mechanistic studies suggest that mutant KBTBD4 neo-substrate interfaces (eg involving HDAC1/2-containing complexes) may represent a more specific therapeutic opportunity than global proteasome inhibition.^26^

In contrast, ATRX-deficient, ALT-positive tumors exhibit selective vulnerability to ATR kinase inhibition. Functional studies have shown that such tumors are hypersensitive to ATR inhibitors such as ceralasertib, and early pediatric clinical evidence, including partial response in pediatric PB treated with ceralasertib-based combinations, provides proof of concept, although translation to PPTID remains investigational.^23^^,^^27^^,^^28^

Unlike PBs, PPTIDs are primarily characterized by *KBTBD4* insertions and *ATRX* loss rather than alterations in small RNA processing pathways.^20^ Integrated exome sequencing and array-based cytogenetic profiling have expanded the mutational spectrum of PPTID beyond canonical *KBTBD4* alterations, identifying additional variants, such as *TSC1* L388P and *IKZF3* F206C, as well as large chromosomal losses involving *PTEN* and *TP53*, implicating dysregulation of cell-cycle control and PI3K/mTOR signaling^29^ (Table 1).

**Figure 1.**
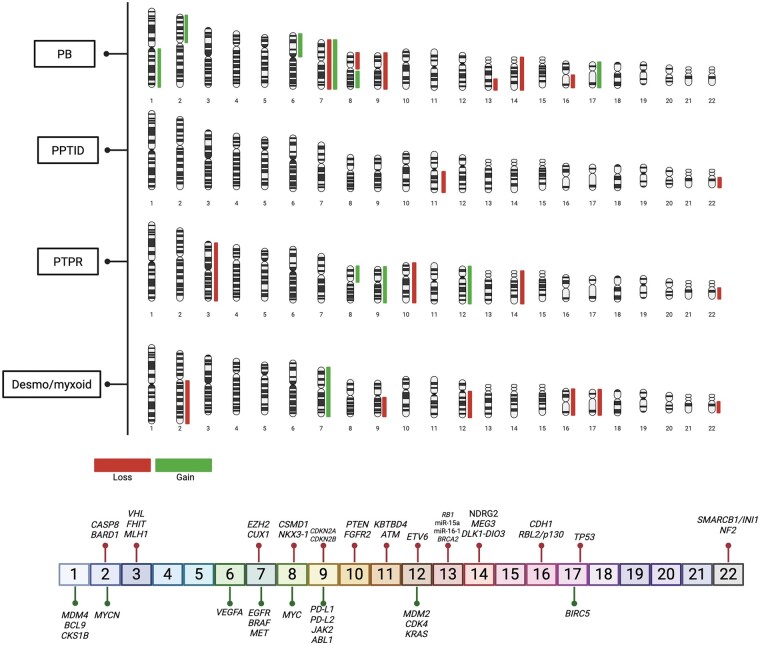
Cytogenetic landscape of pineal parenchymal tumors. Representative chromosomal ideograms illustrating recurrent copy-number alterations (CNAs) across major pineal tumor subtypes, including pineoblastoma (PB), pineal parenchymal tumor of intermediate differentiation (PPTID), papillary tumor of the pineal region (PTPR), and desmoplastic myxoid tumor, SMARCB1-mutant. Distinct subtype-specific patterns of chromosomal gains and losses are observed. Pineoblastomas display recurrent imbalances involving chromosomes 2, 8, 9, 10, 14, and 16.^20^^,^^52^^,^^53^ PPTIDs show heterogeneous genomic profiles with variable focal alterations, including losses of 11q and 22q.^20^^,^^30^ PTPRs are characterized by consistent loss of chromosome 10, frequently encompassing PTEN, together with additional losses of 3p, 14q, and 22q and gains of 8p and 12q.^80^^,^^81^ Desmoplastic myxoid tumors, SMARCB1-mutant, show recurrent loss of 22q involving SMARCB1, in association with additional losses of 3p, 9q, 10q, 13q, and 16q and occasional gains of 8p.^86^ Bars indicate chromosomal gains and losses. Selected oncogenes and tumor suppressor genes mapping to recurrently altered loci are shown below the ideograms.

Cytogenetically, PPTIDs display a heterogeneous profile. Case-level karyotyping and array comparative genomic hybridization (aCGH) studies have identified focal losses involving chromosomes 22q and 11q, as well as occasional large-scale structural rearrangements^20^^,^^30^ (Figure 1). Together with recurrent *KBTBD4* mutations, these findings underscore the mixed biological nature of PPTID.

**Figure 2.**
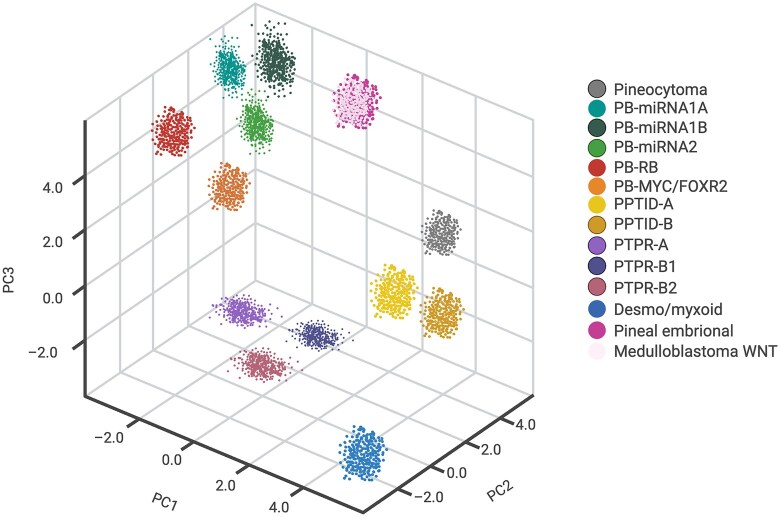
Schematic overview of epigenetic clustering of pineal parenchymal tumors and related embryonal entities. This figure provides an illustrative summary of DNA methylation-based classifications reported in previously published studies.^6^^,^^20^^,^^45^^,^^68^^,^^83^^,^^86^ The depicted clusters are derived from integrated analyses of genome-wide methylation profiles and are intended to summarize established molecular subgroups rather than represent original or newly generated datasets. No primary methylation data were generated for this review. Pineoblastomas segregate into PB-miRNA1A, PB-miRNA1B, PB-miRNA2, PB-RB, and PB-MYC/FOXR2 groups, while pineal parenchymal tumors of intermediate differentiation separate into PPTID-A and PPTID-B subgroups. Papillary tumors of the pineal region further cluster into PTPR-A, PTPR-B1, and PTPR-B2 entities. Pineocytoma and DMT-SMARCB1 form distinct epigenetic clusters, consistent with published classifications. WNT-activated embryonal tumors cluster with WNT-subgroup medulloblastomas, reflecting shared epigenetic features.

Epigenetically, PPTIDs form a recognizable DNA methylation class (Figure 2). Methylation profiling has further refined PPTID by identifying at least 2 epigenetically distinct subgroups (PPTID-A and PPTID-B). One subgroup is characterized by recurrent *KBTBD4* in-frame insertions and represents the molecular core of PPTID, whereas a second, more heterogeneous subgroup lacks *KBTBD4* alterations and may exhibit *ATRX* loss and *ALT* activation, highlighting biological diversity within tumors classified histologically as PPTID.^6^^,^^20^

## Pineoblastoma

Pineoblastoma is a poorly differentiated embryonal neoplasm arising from the pineal parenchyma and represents the most aggressive entity among pineal parenchymal tumors. Craniospinal dissemination is observed in approximately 25%-33% of patients at diagnosis.^31–34^ PBs account for about 35% of all pineal parenchymal tumors^34–38^ and occur predominantly in children, with a reported median age at diagnosis of around 6 years.^39^ Despite multimodal treatment approaches, overall prognosis remains poor, with median overall survival reported in the range of 4.1-8.7 years.^40^^,^^41^ However, both clinical presentation and outcome vary substantially according to underlying molecular features.

- Germline alterations. While most pineal parenchymal tumors are sporadic, a clinically relevant subset of PBs arises in the context of germline cancer predisposition syndromes (Table 1).
- In the WHO 2021 classification of CNS tumors, germline predisposition to pediatric pineal parenchymal tumors, particularly PB, is increasingly recognized, with pathogenic variants most consistently described in the tumor suppressor genes *RB1* and *DICER1* and in the microRNA-processing gene *DROSHA* (Table 1).
- *RB1* encodes the retinoblastoma protein, a key regulator of the G1/S cell-cycle checkpoint through sequestration of E2F transcription factors; germline loss-of-function variants in *RB1* underlie hereditary retinoblastoma and can give rise to trilateral retinoblastoma when PB develops, following the classical 2-hit inactivation model with a germline truncating or splice-site variant followed by somatic loss of the second allele.^42^ These tumors typically present at very young age (2.1 years) and are associated with particularly poor outcomes (median OS 2.8 years).^43^
- In older children, PB has also been associated with loss of function germline alterations in microRNA biogenesis genes, including *DGCR8* (DiGeorge syndrome), *DICER1* (DICER1 tumor predisposition syndrome) and *DROSHA*, as well as alterations in *APC* in the context of Turcot syndrome.^43–46^
- *DICER1*, which encodes a cytoplasmic RNase III endoribonuclease responsible for the processing of precursor microRNAs (pre-miRNAs) into mature, functional miRNAs, represents another major susceptibility gene for PB. Pathogenic germline alterations are typically truncating or frameshift variants involving the RNase IIIb or helicase domains, leading to impaired miRNA cleavage. Tumorigenesis frequently follows a second somatic hit, most commonly loss of heterozygosity.^46^ Alterations in DICER1 have been reported in approximately 15%-20% of PB cases in a single series.^46^
- More recently, *DROSHA*, which encodes the nuclear RNase III endoribonuclease that constitutes the core catalytic component of the microRNA (miRNA) processing machinery, has also been implicated in PB susceptibility. Drosha mediates the initiation step of miRNA biogenesis by cleaving primary miRNA transcripts (pri-miRNAs) into hairpin-shaped precursor miRNAs (pre-miRNAs) within the nucleus, thereby enabling subsequent cytoplasmic processing.^47^ Eight of 9 children from independent families with heterozygous germline pathogenic variants predominantly loss-of-function developed PB of the miRNA processing altered_1 subtype, with each tumor exhibiting a somatic second hit, consistent with a recessive mechanism.^47^
- *DGCR8* is a double-stranded RNA-binding protein and, together with the RNase III enzyme Drosha, forms the nuclear Microprocessor complex, which recognizes and cleaves primary miRNA transcripts. The 22q11.2 deletion syndrome (DiGeorge syndrome) is one of the most common recurrent microdeletion syndromes, caused by a heterozygous deletion encompassing multiple genes, including *DGCR8*, and characterized by variable congenital, immunological, and neurodevelopmental features. Loss of one *DGCR8* allele leads to defective miRNA biogenesis, providing a plausible biological mechanism linking this syndrome to tumor predisposition, including rare cases of PB.^48^^,^^49^
- Rare additional predisposition syndromes have been described, including a single pediatric case of PB associated with Sotos syndrome caused by a de novo germline loss-of-function variant in *NSD1*, encoding a histone lysine methyltransferase, although evidence remains limited to this isolated report.^50^
- In a large retrospective Chinese cohort, germline pathogenic variants were identified across 34 genetic tumor syndromes associated with CNS tumors, including pineal region tumors, with *TP53, MSH2, NF1*, and *BRCA2* being the most frequently affected genes.^51^
- In line with WHO CNS 2021 recommendations, genetic counseling and germline testing should be considered in all patients with PB, especially in pediatric patients with early age at diagnosis, regardless of family history.^43^
- Somatic alterations. Integrated molecular profiling has demonstrated that PB is a biologically heterogeneous disease and has enabled its subdivision into 4 major molecular subgroups with distinct genetic drivers and clinicopathological features: miRNA processing-altered_1A and B (PB-miRNA1A and B), miRNA processing-altered_2 (PB-miRNA2), RB1-altered (PB-RB1), and MYC/FOXR2-activated (PB-MYC/FOXR2) tumors^20^^,^^45^ (Table 1).
- Tumor groups showed significant differences in age at diagnosis, sex predilection, and metastatic status.^52^
- MiRNA-altered PB predominantly occurred in pediatric patients, with children in the miRNA1 group being younger than those in the miRNA2 group. While miRNA1 tumors were associated with intermediate outcomes, PB-miRNA2 tumors demonstrated favorable survival similar to PPTID. In contrast, PB-MYC/FOXR2 and PB-RB1 subtypes were primarily observed in very young children and were characterized by aggressive clinical behavior and dismal prognosis.^52^
- PB-miRNA subtypes are characterized by mutually exclusive inactivating alterations in *DROSHA, DICER1,* or *DGCR8*. These alterations predominantly result in biallelic loss of function, achieved through homozygous deletions, compound heterozygous truncating mutations, or a combination of mutation and copy-number loss. Missense variants are rare. *DGCR8* alterations are restricted to PB-miRNA1A, whereas *DROSHA* and *DICER1* alterations occur across all miRNA-altered subtypes.^52^^,^^53^
- Additional *BCOR* variants were identified in a PB-miRNA2 tumor with homozygous *DROSHA* deletion and in a PB-miRNA1A tumor with a heterozygous DROSHA mutation, supporting the notion that these alterations may represent secondary or passenger events.^45^  *BCOR* is a transcriptional repressor, altered in various solid and hematologic malignancies, including acute myeloid leukemia.^54^
- Emerging evidence indicates that tumorigenesis in PBs may be driven primarily by recurrent copy-number alterations rather than point mutations.^55^ In particular, whole-genome sequencing analyses have identified copy-number gains of the *PDE4DIP* gene (myomegalin) in primary tumors and metastatic lesions, suggesting a potential role in tumor progression.^55^ More recent data reveal recurrent duplications of *PDE4DIP*, especially enriched in the PB-RB1 and PB-MYC/FOXR2 subtypes. Given that these molecular groups are associated with pronounced genomic instability and poor clinical outcome, *PDE4DIP* copy-number gains may represent a marker of aggressive disease biology and unfavorable prognosis.^52^ Beyond the miRNA-altered PB subtypes, distinct genetic drivers characterize the remaining groups. PB-MYC/FOXR2 tumors lack pathogenic single-nucleotide variants and are instead defined by focal *MYC* amplification, whereas PB-RB1 tumors predominantly exhibit biallelic *RB1* inactivation at chromosome 13q14.2 through focal loss and/or truncating mutations.^52^
- Cytogenetic alterations. The PB-miRNA1 group exhibited the highest burden of chromosomal alterations among all PB subgroups, with PB-miRNA1B showing a greater number of events compared with PB-miRNA1A. Both subgroups were characterized by multiple polyploidy events, indicating marked chromosomal instability.^20^^,^^52^^,^^53^ A recurrent feature shared by PB-miRNA1A and PB-miRNA1B tumors was whole chromosome 14 loss, representing a common cytogenetic hallmark of this group. In addition, PB-miRNA1B tumors were further distinguished by the frequent loss of chromosome 16, highlighting a more complex and unstable genomic profile compared with PB-miRNA1A.^52^,^53^
- The PB-miRNA2 subgroup also displayed recurrent chromosome 14q or whole chromosome 14 loss, which was consistently associated with point mutations in *DICER1.*^45^ In contrast to PB-miRNA1 tumors, PB-miRNA2 samples showed the lowest overall level of chromosomal alterations, suggesting a more genomically stable background.^20^^,^^52^^,^^53^ Despite this relative stability, recurrent whole chromosome 7 loss and chromosome 9 loss were observed, defining a distinct cytogenetic pattern for this subgroup^52^^,^^53^ (see Figure 1).
- In contrast to the miRNA-driven subgroups, the PB-MYC/FOXR2 subtypes displayed a lower burden of whole-chromosome alterations compared with PB-miRNA1, while still occupying an intermediate level of cytogenetic complexity within the PB spectrum.
- The PB-MYC/FOXR2 subgroup was characterized by recurrent whole chromosome gains, including chromosome 1 gain and chromosome 7 gain, as well as 8q gain encompassing the MYC locus, accompanied by 8p loss. Additional recurrent alterations included chromosome 17 gain with the formation of an isochromosome i(17q), polyploidy events, and 16q loss. Overall, the copy number landscape of PB-MYC/FOXR2 tumors was dominated by chromosomal gains, suggesting that increased gene dosage represents a major oncogenic mechanism in this subgroup^20^,^52^,^53^ (Figure 1).
- The PB-RB1 subgroup exhibited a low level of whole-chromosome alterations but with highly specific and recurrent cytogenetic changes. These included whole chromosome 16 loss or 16q loss, 6p gain, 1q gain, and 2p gain. Importantly, focal loss of 13q31.1, corresponding to the RB1 locus, was consistently observed, together with gain of the miR-17/92 cluster located at the terminal region of chromosome 13q.^20^^,^^52^^,^^53^ This pattern highlights a targeted disruption of cell cycle regulation in the context of an otherwise relatively stable chromosomal background.
- Functional mechanisms involved. These genetic alterations converge on a limited number of functional pathways, most notably microRNA biogenesis, cell-cycle regulation, and transcriptional control. In PB-miRNA subgroups, disruption of microRNA production represents a central oncogenic mechanism. Core components of the miRNA biogenesis machinery, including *DICER1*, *DROSHA*, and *DGCR8*, are recurrently altered, leading to a global reduction in mature miRNA levels.^45^^,^^46^^,^^55^
- Functionally, hotspot mutations affecting the RNase IIIb domain of DICER1 selectively impair the processing of 5p-derived miRNAs, resulting in a pronounced depletion of tumor-suppressive miRNAs such as members of the let-7 family. Loss of let-7–mediated repression leads to derepression of oncogenic targets, including PLAGL2, thereby promoting progenitor-like transcriptional programs and sustained cellular proliferation.^56^ This mechanism is further supported by experimental evidence from mouse models, in which conditional ablation of Drosha or Dicer1 in the pineal lineage results in accelerated S-phase entry and the development of PB-like tumors, directly linking impaired miRNA-mediated post-transcriptional regulation to tumor initiation and growth.^57^^,^^58^
- In PB-RB1 tumors, biallelic loss of *RB1* results in constitutive activation of E2F transcription factors, removing a central checkpoint that normally restrains G1/S transition. This alteration frequently co-occurs with focal copy-number gains of the miR-17∼92 cluster (MIR17HG, 13q31.3), leading to overexpression of miR-17-5p, miR-18a, miR-19a/b, and miR-92a. These miRNAs act post-transcriptionally to reinforce E2F-driven cell-cycle programs and to attenuate compensatory cell-cycle inhibitory responses, including repression of CDKN1A/p21, thereby stabilizing sustained S-phase entry.^58^ Together, RB1 loss and miR-17∼92 upregulation establish a proliferative state that closely phenocopies the core biology of retinoblastoma.^58^ In this context, pharmacologic inhibition of CDK4/6 is unlikely to restore cell-cycle control, as these agents require functional RB1 to restrain E2F activity, providing a mechanistic explanation for the limited efficacy of CDK4/6 inhibitors observed in this subgroup.^59^
- A third oncogenic axis is represented by PB-MYC/FOXR2, in which MYC amplification together with aberrant FOXR2 activation drives uncontrolled transcriptional amplification; FOXR2 stabilizes MYC protein and augments MYC/MAX E-box-dependent programs governing ribosome biogenesis, nucleotide metabolism, and cell-cycle progression, establishing a feed-forward circuit consistent with transcriptional addiction described in other embryonal brain tumors.^60–63^
- In this context, preclinical studies have shown that BET bromodomain inhibitors and CDK9 inhibitors can attenuate MYC-dependent transcription, nominating this pathway as an attractive, though still experimental, therapeutic target.^64–66^
- Transcriptomic signature. Transcriptomic profiling demonstrates that pineal parenchymal tumors are characterized by distinct and reproducible gene expression programs that closely reflect their underlying molecular drivers. Unsupervised analyses based on highly variable genes robustly separate PBs into discrete subgroups, including miRNA-driven tumors, MYC/FOXR2-driven tumors, RB1-deficient tumors, and PPTID, indicating profound transcriptional divergence among these entities.^52^ At a global level, PBs exhibit a transcriptional identity clearly distinct from non-pineal parenchymal tumors, defined by coordinated deregulation of genes involved in neurodevelopmental processes and pineal lineage specification.
- A central component of this lineage-specific program is the sustained expression of the homeobox transcription factors CRX and OTX2, which are key regulators of pinealocyte and photoreceptor differentiation during development. CRX functions as a master transcriptional regulator of pineal and retinal lineage genes, controlling programs related to phototransduction, neuronal differentiation, and circadian biology, while OTX2 acts upstream in pineal and retinal development, maintaining progenitor identity and regulating CRX-dependent transcriptional networks. The elevated expression of CRX observed across miRNA-driven PBs likely reflects retention of a pinealocyte-like differentiation state, whereas its reduction in non-miRNA-driven tumors suggests partial loss of lineage commitment.
- Similarly, OTX2 expression is highest in PB-miRNA1 tumors and progressively decreases in PB-miRNA2 and MYC/FOXR2-driven tumors, indicating attenuation of pinealocyte lineage features in these subgroups and a shift toward alternative oncogenic transcriptional programs.^52^^,^^53^ In contrast, RB1 expression is selectively reduced in RB1-driven PBs, consistent with biallelic gene inactivation, while FOXR2 is specifically and markedly overexpressed in MYC/FOXR2-driven tumors, confirming its role as a defining transcriptional driver and reflecting MYC-dependent transcriptional amplification.^52^^,^^53^
- Methylation profile. DNA methylation profiling has therefore profoundly reshaped classification of PB, such that in the WHO 2021 framework these tumors are no longer regarded as a homogeneous entity but instead segregate into 5 reproducible epigenetic subgroups corresponding to PB-miRNA1A/B, PB-miRNA2, PB-RB, and PB-MYC/FOXR2 classes, which are clearly distinct from other brain tumor histotypes^45^ (Figure 2). Beyond the well-defined PB subgroups, a small subset of embryonal tumors arising in the pineal region has been identified that does not cluster with canonical PBs on DNA methylation profiling. In a multi-institutional cohort of seven patients, Liu and colleagues described embryonal tumors of the pineal region that, despite histological features compatible with PB or other CNS embryonal tumors, consistently aligned with the WNT-activated medulloblastoma subgroup on DNA methylation analysis. These tumors share hallmark molecular features of WNT pathway activation, including canonical CTNNB1 exon 3 mutations, nuclear β-catenin accumulation, *LEF1* expression, and frequent monosomy 6. Clinically, affected patients were predominantly adolescents presenting with localized disease, and all were alive at a median follow-up of three years, suggesting a favorable prognosis comparable to that of posterior fossa WNT medulloblastoma. Although these tumors are not currently recognized as a distinct entity in the WHO 2021 classification, accumulating evidence indicates that WNT-driven embryonal tumors of the pineal region warrant further investigation. Their biological interpretation remains debated: they may represent either an ectopic manifestation of medulloblastoma biology arising in the pineal region or a novel, molecularly defined embryonal tumor subtype within the pineal tumor spectrum that converges epigenetically and biologically with WNT medulloblastoma despite its atypical anatomical location.^67^^,^^68^
- Target therapy. Targeted therapy in PB remains challenging because most studies enroll PB within broader pediatric CNS tumor cohorts rather than PB-specific molecularly stratified trials. Nonetheless, several molecular inhibitors have been explored in relapse settings or in biomarker-driven contexts, including multi-kinase inhibition (dasatinib), PI3K/AKT/mTOR pathway blockade (sirolimus/everolimus), and tumor-agnostic TRK inhibition in rare NTRK fusion-positive cases, while transcriptional/epigenetic strategies (eg BET or HDAC inhibition) are emerging for MYC-driven biology.^69^

## Papillary Tumor of the Pineal Region

Papillary tumor of the pineal region is a rare neuroepithelial neoplasm characterized by a distinctive combination of papillary architecture and epithelial-like tumor cells, arising specifically within the pineal region. According to the current CNS WHO classification, PTPRs are assigned a WHO grade 2 or 3, with the majority of cases corresponding to grade 2.^45^ Clinically, PTPRs are notable for a high rate of local recurrence, and spinal dissemination has been reported in a subset of cases. PTPRs occur across a broad age range, affecting both children and adults. In a multicenter study, the estimated 5-year overall survival and progression-free survival rates were 73% and 27%, respectively, with incomplete surgical resection being significantly associated with increased risk of recurrence and reduced survival.^70–72^

- Germline Alterations. To date, the genetic etiology of PTPR remains largely unknown, and no recurrent germline predisposition has been established (Table 1). Accordingly, the WHO classification does not currently associate PTPR with a defined hereditary cancer predisposition syndrome.^43^
- Emergent evidence has begun to describe a germline association in a subset of cases. In a first report, a 21-year-old male presenting with macrocephaly and obstructive hydrocephalus was diagnosed with a PTPR. Tumor sequencing revealed a pathogenic PTEN p. Gly132Asp (p.G132D) variant, and matched germline testing confirmed the presence of PTEN hamartoma tumor syndrome (PHTS).^73^ PHTS is a rare autosomal dominant cancer predisposition disorder caused by germline pathogenic variants in the PTEN tumor suppressor gene, which encodes a lipid phosphatase that negatively regulates the PI3K-AKT-mTOR signaling pathway. PHTS encompasses a spectrum of clinical entities, including Cowden syndrome and Lhermitte-Duclos disease (OMIM#158350) and related phenotypes, and is characterized by macrocephaly, mucocutaneous hamartomas, developmental abnormalities, and an increased lifetime risk of multiple malignancies, including malignant lesions of the CNS.^74^^,^^75^ More recently, germline sequencing has identified constitutional heterozygous pathogenic or likely pathogenic (P/LP) PTEN variants in 5 individuals with PTPR, substantially expanding the evidence for a germline contribution in a subset of cases. Identified variants were predominantly truncating (n = 4), with one missense variant affecting the phosphatase domain (n = 1). Patients harboring germline PTEN variants were significantly younger at diagnosis compared with those without germline alterations (mean age 2.2 vs 14.2 years, p = 0.014), and all affected individuals were under 5 years of age at presentation.^76^ These findings suggest that germline PTEN alterations may define a distinct, early-onset subset of PTPR, with important implications for genetic counseling, clinical surveillance, and tumor classification.
- Somatic alterations. The most characteristic somatic alterations in PTPR involve the PTEN gene on chromosome 10 (Table 1). These alterations predominantly consist of loss-of-function events, including truncating mutations and focal deletions, frequently affecting exon 7, with recurrent variants such as G251D and Q261*.^77^ These mutations disrupt the region of PTEN encoding the C2 domain, which is critical for PTEN-membrane interaction and for positioning the catalytic site at the plasma membrane to enable dephosphorylation of membrane-bound PIP3. Accordingly, the frequent loss of the PTEN locus is likely to play a central role in PTPR pathogenesis. Functional inactivation of the PTEN tumor suppressor results in constitutive activation of the PI3K/AKT/mTOR signaling pathway, providing a biological rationale for the potential clinical use of PI3K/AKT/mTOR pathway inhibitors, such as everolimus, as a therapeutic option in this tumor entity.^78^^,^^79^
- Cytogenetic alterations. At the cytogenetic level, PTPR are characterized by recurrent somatic copy number alterations involving multiple chromosomes. Loss of chromosome 10 is a frequent event and often encompasses the PTEN locus, further supporting the central role of PTEN inactivation in tumor development. Additional recurrent chromosomal losses include chromosome 3, chromosome 14, and chromosome 22, with either 22q-specific loss or whole chromosome 22 loss observed in a subset of cases. Conversely, recurrent chromosomal gains have been reported on chromosome 8p, as well as on chromosomes 9 and 12 (Figure 1). Together, these cytogenetic alterations point to a pattern of widespread genomic imbalance in PTPR, likely contributing to tumor initiation and progression through combined loss of tumor suppressor genes and gain of oncogenic drivers.^80^^,^^81^
- Expression profile. Gene expression profiling has shown that PTPR express several genes known to be highly enriched in the rodent subcommissural organ (SCO), including CALCA (encoding calcitonin-related polypeptide alpha), FERD3L (Fer3-like basic helix-loop-helix transcription factor), and SPDEF (SAM-pointed domain–containing ETS transcription factor), supporting a developmental relationship with SCO-derived lineages rather than with pineal parenchyma.^81^ Also FOXJ1/CRX immunohistochemistry supports an ependymal/SCO-like phenotype in PTPR: normal pineal tissue is diffusely CRX-positive and FOXJ1-negative, whereas PTPR shows diffuse nuclear FOXJ1 with absent or only focal/rare-cell CRX, mirroring fetal SCO (FOXJ1-positive, CRX-negative). Thus, the FOXJ1-CRX panel helps distinguish PTPR from pineal parenchymal tumors among non-germ cell pineal region neoplasms.^82^
- Methylation profile. DNA methylation profiling has emerged as a key tool for the molecular classification of PTPR. As previously reported, DNA methylation-based clustering identifies two major methylation groups, termed PTPR-A and PTPR-B (Figure 2). Subsequent integrative analyses combining methylation data with genomic copy number profiles have further refined this classification, allowing subdivision of the PTPR-B group into PTPR-B1 and PTPR-B2 subtypes. Notably, recurrent losses of chromosome 3 or chromosome 14 are characteristic of PTPR-B1 tumors, whereas these alterations are not observed in PTPR-B2, supporting biological heterogeneity within the PTPR-B group.^20^^,^^83^
- Target therapy. Given the frequent chromosome 10 loss and recurrent PTEN alterations in PTPR, activation of the PI3K/AKT/mTOR axis represents a plausible therapeutic vulnerability. Clinical evidence remains limited to case-based reports; however, durable radiographic responses to mTORC1 inhibition with everolimus have been described, including a PTEN-mutant PTPR treated with everolimus monotherapy and additional reports in the relapsed setting. While prospective PTPR-specific trials are lacking, these observations provide a rationale for considering mTOR inhibition in recurrent or progressive PTEN-altered PTPR within a molecularly informed treatment framework.^84^^,^^85^

## Desmoplastic Myxoid Tumor

Desmoplastic myxoid tumor of the pineal region, SMARCB1-mutant is a recently described entity characterized by prominent myxoid stromal changes and desmoplasia in the absence of overt histopathological features of high-grade malignancy. Reported cases are rare and predominantly affect adults, with a median age at diagnosis of approximately 40 years among the 7 cases described to date. Clinically, these tumors appear to follow a less aggressive course than atypical teratoid/rhabdoid tumors (AT/RT), despite sharing alterations in SMARCB1. No evidence of metastatic dissemination has been documented at presentation or during follow-up. However, the biological behavior remains incompletely defined, as tumor grade has not yet been formally assigned, and disease-related mortality has been observed in 3 of 7 patients after a median follow-up of approximately 48 months, underscoring the need for additional cases and longer follow-up to better define prognosis and optimal management.^43^^,^^86^

- Somatic alterations. Molecular genetic examinations revealed recurrent *SMARCB1* alterations in desmoplastic myxoid tumor of the pineal region. Specifically, heterozygous *SMARCB1* deletions were identified in three cases, while homozygous deletions were detected in 2 additional cases (Table 1). Targeted sequencing demonstrated further disruption of the remaining allele in individual tumors, including a 2-bp deletion (c.369_370del) resulting in a frameshift mutation (p.Lys124Glyfs*45) in 1 case and a homozygous duplication in exon 3 (c.237_276dup) leading to a frameshift mutation (p.Ala93Argfs*26) in another, consistent with biallelic inactivation of *SMARCB1*. In contrast, *EWSR1* rearrangements, previously described in intracranial extraskeletal myxoid chondrosarcomas, were not detected in the 2 cases, supporting the molecular distinction of this tumor entity.^86^^,^^87^  *SMARCB1* encodes a core subunit of the SWI/SNF (BAF) chromatin-remodeling complex, which normally antagonizes PRC2/EZH2-mediated transcriptional repression. Loss of SMARCB1 results in functional inactivation of the SWI/SNF complex, leading to epigenetic imbalance characterized by increased PRC2 dependency and aberrant H3K27 trimethylation. This acquired reliance on EZH2 activity represents a well-established synthetic vulnerability of SMARCB1-deficient tumors.^88^
- Cytogenetic alterations. The most consistent abnormality was loss of chromosome 22q, observed as a focal deletion in the majority of evaluable cases, in keeping with involvement of the *SMARCB1* locus. Additional recurrent alterations included focal losses on chromosome arms 2q, 12q, 16, and 17, as well as loss of the entire chromosome 9q in a subset of tumors. In contrast, chromosomal gains were uncommon, with gain of whole chromosome 7 identified in a single case (Figure 1). Overall, the cytogenetic landscape is characterized by predominantly focal deletions rather than widespread chromosomal instability, supporting a model in which targeted loss of tumor suppressor regions most notably chromosome 22q plays a central role in tumor pathogenesis.^86^
- Methylation profile. DNA methylation–based t-SNE analysis demonstrates that desmoplastic myxoid tumor of the pineal region, SMARCB1-mutant forms a distinct and compact methylation cluster, clearly separated from established CNS tumor entities (Figure 2). In particular, these tumors segregate away from the major AT/RT methylation subgroups (ATRT-MYC, ATRT-SHH, and ATRT-TYR), as well as from poorly differentiated chordoma and neural malignant rhabdoid tumor (MRT). The clear spatial separation on the t-SNE plot supports the interpretation that desmoplastic myxoid tumor of the pineal region represents a molecularly distinct epigenetic entity, despite sharing *SMARCB1* alterations with AT/RT, and argues against classification within existing AT/RT methylation classes.^86^
- Target therapy. DMT of the pineal region is defined by SMARCB1/INI1 loss, suggesting a biologically grounded vulnerability to PRC2/EZH2 dependency. Accordingly, EZH2 inhibition has emerged as a rational therapeutic strategy in this molecular context. The selective EZH2 inhibitor tazemetostat has demonstrated clinical activity in SMARCB1-deficient malignancies, including epithelioid sarcoma and pediatric rhabdoid tumors, and has been evaluated in pediatric CNS cohorts, supporting PRC2 blockade as the most plausible targeted therapeutic avenue for SMARCB1-mutant pineal region tumors. However, dedicated clinical evidence in DMT-SMARCB1 is currently lacking, and targeted therapy remains extrapolative rather than established.^89^^,^^90^

## Conclusions

**Figure 3.**
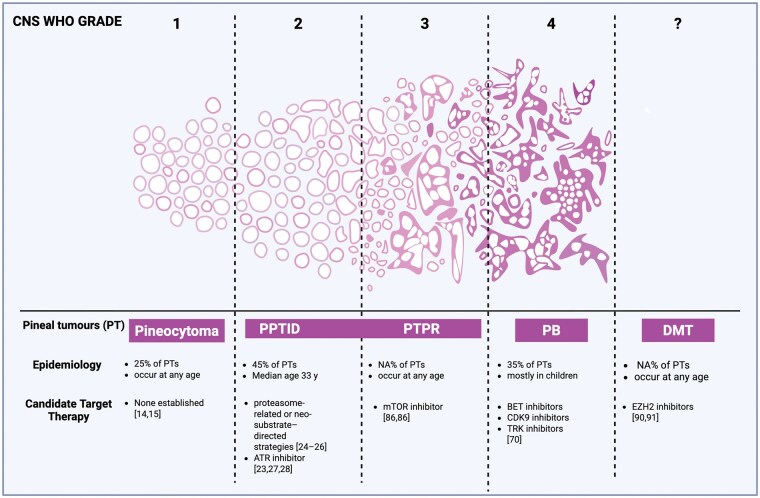
Spectrum of pineal region tumors across WHO grades, epidemiological features, and candidate targeted therapies. Schematic overview of pineal parenchymal tumor entities according to CNS WHO grade and epidemiological characteristics. The figure summarizes pineocytoma, pineal parenchymal tumor of intermediate differentiation (PPTID), papillary tumor of the pineal region (PTPR), pineoblastoma (PB), and desmoplastic myxoid tumor, SMARCB1-mutant (DMT), together with currently proposed candidate targeted therapies derived from published preclinical and clinical studies.

Pineal region tumors comprise a rare and biologically diverse group of CNS neoplasms whose clinical behavior is increasingly understood through integrated molecular profiling rather than histology alone. Advances in genomics, epigenetics, and transcriptomics have revealed that pineal parenchymal tumors exist along a continuum of molecularly defined entities, each driven by distinct oncogenic mechanisms and associated with specific prognostic and therapeutic implications, as summarized in Figure 3.

## Data Availability

No new data were generated in this study. All data are derived from previously published studies.
